# Application of 3-dimensional visualization and image fusion technology in liver cancer with portal vein tumor thrombus surgery

**DOI:** 10.1097/MD.0000000000038980

**Published:** 2024-07-26

**Authors:** Yong Tan, Jian Yong Zhu, Jing Li, Li Ming Wu, Zaixing Ouyang, Wen Ying Liu, Hao Song, Cong Yun Huang

**Affiliations:** a Department of Hepatobiliary Surgery, Yuebei People’s Hospital, Shaoguan, P.R. China; b Senior Department of Hepato-Pancreato-Biliary Surgery, the First Medical Center of PLA General Hospital, Beijing, P.R. China; c Department of Pathology, Yuebei People’s Hospital, Shaoguan, P.R. China.

**Keywords:** 3-dimensional visualization, image fusion, liver cancer, portal vein tumor thrombus, surgery

## Abstract

Liver cancer with portal vein tumor thrombus (PVTT) is a frequent finding and is related to poor prognosis. Surgical resection provides a more promising prognosis in selected patients. The purpose of this study was to explore the application of 3D (3-dimensional) visualization and image fusion technology in liver cancer with PVTT surgery. 12 patients were treated with surgery between March 2019 and August 2022. The preoperative standard liver volume (SLV), estimated future liver remnant (FLR), FLR/SLV, 3D visualization models, PVTT classification, operation programs, surgical results, and prognosis were collected and analyzed. Twelve patients who had complete data of 3D visualization and underwent hemihepatectomy combined with portal vein tumor thrombectomy. The operation plan was formulated by 3D visualization and was highly consistent with the actual surgery. The SLV was 1208.33 ± 63.22 mL, FLR was 734.00 mL and FLR/SLV was 61.62 ± 19.38%. The accuracy of classification of PVTT by 3D visualization was 100%, Cheng type Ⅱa (4 cases), Ⅱb (2 cases), Ⅲa (4 cases), and Ⅲb (2 cases). The 3D visualization model was a perfect fusion with the intraoperative live scene and precise guidance for hepatectomy. No patient was suffering from postoperative liver failure and without procedure‑associated death. 6 patients died of tumor recurrence, and 2 patients died of other reasons. The 12-month cumulative survival rate was 25.9%. 3D visualization and image fusion technology could be used for precise assessment of FLR, classification of PVTT, surgery navigation, and which was helpful in improving the safety of hepatectomy.

## 1. Introduction

Primary liver cancer remains a common human malignancy and is extremely responsible for threatening human health. Global Cancer Statistics 2020 estimated that primary liver cancer was the sixth most common human cancer and the third leading cause of cancer death worldwide, accounting for 905,677 new cases and 830,180 deaths.^[1]^ The overwhelming majority of patients with primary liver cancer had been diagnosed in the advanced stage and were considered incurable. Portal vein tumor thrombus (PVTT) formation is a characteristic progression pattern of primary liver cancer, especially hepatocellular carcinoma. For these patients the median survival time was 2.7 months if untreated, and only slightly longer after systemic therapy, such as tyrosine kinase inhibitors.^[2]^ Surgery resection was justified as the last chance. An aggressive surgical strategy could benefit patient survival in selected cases, not only improving the quality of life but also prolonging the survival time.^[2,3]^ Peng et al reported that surgical treatment for liver cancer patients with PVTT achieved good results with the longest surviving time being 13 years and onward.^[3]^ However, surgical treatment of primary liver cancer with PVTT is one of the thorniest questions in liver surgery.

Precise liver resection is a new liver surgery conception and technology system in the new century. It breaks through the limitations of traditional liver resection, relying on the system of “visualization, quantification and controllability,” and realizing liver surgery is “safe, efficient and minimally invasive.”^[4]^ Nevertheless, traditional two-dimensional (2D) images have been unable to meet the requirements of precise resection in liver surgery because of their limitations in preoperative evaluation and real-time surgical guidance. The emergence of 3-dimensional (3D) visualization, 3D printing, and image fusion technology provides more comprehensive individualized anatomical information on the liver for clinical surgeons and thus improves the safety and precision of liver surgery.^[5–10]^

Our team applied digital medical imaging technology to liver surgery over a period of approximately 15 years of experience and achieved good results. Consequently, 12 cases of primary liver cancer with PVTT whose clinical and digital imaging data between March 2019 and August 2022 were analyzed retrospectively. The purpose of this paper was to explore the value of 3D visualization and image fusion technology in the surgical treatment of primary liver cancer with PVTT.

## 2. Materials and methods

The study was conducted in conformity with the Declaration of Helsinki. Ethical approval was obtained from the ethics committee (Approval No. KY-2018-207). Written informed consent was obtained from all participants.

### 2.1. Patient selection

Primary liver cancer patients with PVTT were treated with surgery between March 2019 and August 2022, and analyzed retrospectively in the present study using a digital medical image database. Inclusive criteria: The patients over 18 years old and preoperative liver function Child-Pugh Grade A; There were complete data of 3D visualization, 3D printing, and intraoperative image fusion. Exclusion criteria: Cheng type Ⅰ, and IV or Japanese Vp classifications Vp1 and Vp2; The patient with Cheng type Ⅱa or Japanese Vp classification Vp3 who did not need portal vein tumor thrombectomy; Previous history of other cancers; Preoperative systemic anticancer therapy or local radiotherapy.

### 2.2. 3D visualization, 3D printing, and image fusion

Patients underwent contrast-enhanced computed tomography (CT) of the liver to obtain 2D images by GE Healthcare Bio-Sciences (Pittsburgh, PA). The 1mm slice thickness images in the plain scan phase, arterial phase, portal venous phase, and venous phase were collected and stored in DICOM format. The DICOM image data were led into 3d-doctor 4.0 software for 3D reconstruction (Trial Version, Able Software Corp), to which it built a 3D visualization image of the liver, tumor, blood vessels, biliary tract, and tumor thrombus. Then, the 3D visualization images were outputted as Standard Template Library files (STL) and imported into Raise 3D software (Shanghai Fuzhi Information Technology Co., Ltd, Shanghai, China). The Raise 3D printer (Shanghai Fuzhi Information Technology Co., Ltd, Shanghai, China) was used to manufacture the models at a ratio of 1:1 or 1:0.7, and the material was polylactic acid (Shenzhen, China). Various functions included showing or hiding different structures and rotating the model to detail understand the anatomic characteristics of the tumor, blood vessels, and PVTT.

In 2012, we began to study polymorphic fusion technology. First, with the help of a projector, the 3D reconstructed image is projected onto the surface of the liver, but the general projector lumens are not enough, and the fusion effect is not very satisfactory. To compensate for this shortcoming, we achieve intraoperative image and 3D visualization image fusion on the computer or smartphone. We are quite satisfied with the accuracy, and the fusion can be completed in 3 to 5 minutes. The main technique is to perform perfect fusion through the midpoint of the gallbladder bed, the superior and inferior hepatic vena cava, the hepatic point near the round ligament of the liver, or the left lateral hepatic margin.

### 2.3. Main outcome measures

Whether the actual operative program was consistent with the simulation surgery plan. Perioperative clinical parameters were collected, which included liver function indexes, preoperative standard liver volume (SLV),^[11]^ estimated future liver remnant (FLR), FLR/SLV, operation time, intraoperative blood loss, pathological results, postoperative hospitalization, procedure‑associated death, perioperative complications and survival time. Liver function indexes were repeated on the first and seventh days after operation, including alanine aminotransferase (ALT), total bilirubin (TB), aspartate aminotransferase (AST), prothrombin time (PT), albumin (ALB). Perioperative main complications: bile leakage, ascites, and abdominal infection.

### 2.4. Follow-up

All patients were followed up by telephone, outpatient clinic, and hospitalization records, including the process of postoperative systemic anti-tumor treatment and survival time. The time of follow-up began on the day of operation and ended in November 2022.

### 2.5. Statistical analysis

All data analyses were performed using SPSS 13.0 statistical software (SPSS, Inc., Chicago, IL). The mean ± standard deviation was used to the measurement data with normal distribution, and the median was applied to the measurement data with skewed distribution. Paired t-test was used for measurement data with normal distribution, and the Wilcoxon rank sum test for measurement data does not obey normal distribution. Survival analysis was calculated using the Kaplan–Meier method from the time of surgery to November 2022. *P* < .05 was considered statistically significant.

## 3. Results

### 3.1. Patient characteristics

A total of 12 patients were enrolled, including 3 women and 9 men. All patients had a background history of hepatitis B virus and received antiviral treatment before the operation. The age was 50.33 ± 7.60 years (range, 39–63 years), and the maximum cross-sectional diameter of the tumor was 10.15 ± 3.83 cm (range, 6.00–17.88 cm).

Preoperative evaluation and surgery navigation assisted by 3D visualization and image fusion 12 patients completed 3D visualization and printed 3D models, which displayed the anatomical structure of the liver, such as the tumor, the vascular system, and the invasion range of PVTT. The PVTT was according to the Cheng or Japanese Vp classification.^[3]^ The classification of preoperative PVTT was precise anastomosis with actual surgery. 3D visualization and 3D printing models can be used to simulate surgery that could determine the hepatectomy plane and the scheme of the tumor thrombectomy. The SLV (1208.33 ± 63.22 mL), FLR (734.00 mL), and FLR/SLV (61.62 ± 19.38%) were calculated before operation (Table 1). All patients were based on the principle that preserved blood vessels of the inflow vasculature and outflow vasculature of FLR so that it prevented ischemia and congestion of FLR.

**Table 1:** Clinical data of liver cancer patients with PVTT.

NO.	Tumor size (cm)	Tumor location	PVTT type	SLV (mL)	FLR (mL)	FLR/SLV (%)	Systemic therapy	Survival time, mo	Pathology
1	7.00	Left liver	Ⅲb/Vp4	1250	990	79.2	-	11 (die)	HCC
2	12.5	Right liver	Ⅱa/Vp3	1242	508	40.9	Sorafenib	30 (survival)	HCC
3	8.0	Right liver	Ⅲb/Vp4	1165	491	42.1	-	5 (die)	HCC
4	7.20	Left liver	Ⅲa/Vp4	1124	487	43.3	-	5 (die)	HCC
5	8.80	Right liver	Ⅱa/Vp3	1263	670	83	Sorafenib	11 (die)	HCC
6	10.20	Left liver	Ⅲa/Vp4	1263	724	57.3	-	17 (die)	HCC
7	17.88	Left liver	Ⅱb/Vp4	1258	764	60.7	-	12 (die)	ICC
8	13.20	Right liver	Ⅱa/Vp3	1186	563	47.4	-	3 (survival)	HCC
9	6.00	Left liver	Ⅲa/Vp4	1305	1411	108.1	Lenvatinib	4 (survival)	HCC
10	8.90	Right liver	Ⅲa/Vp4	1192	792	66.4	Lenvatinib	5 (survival)	HCC
11	15.60	Right liver	Ⅱb/Vp4	1126	844	74.9	-	2 (die)	HCC
12	6.52	Left liver	Ⅱa/Vp3	1126	744	66.1	Lenvatinib	10 (die)	HCC

FLR = future liver remnant, HCC = hepatocellular carcinoma, ICC = intrahepatic cholangiocarcinoma, PVTT = portal vein tumor thrombus, SLV = standard liver volume.

**Figure 1.**
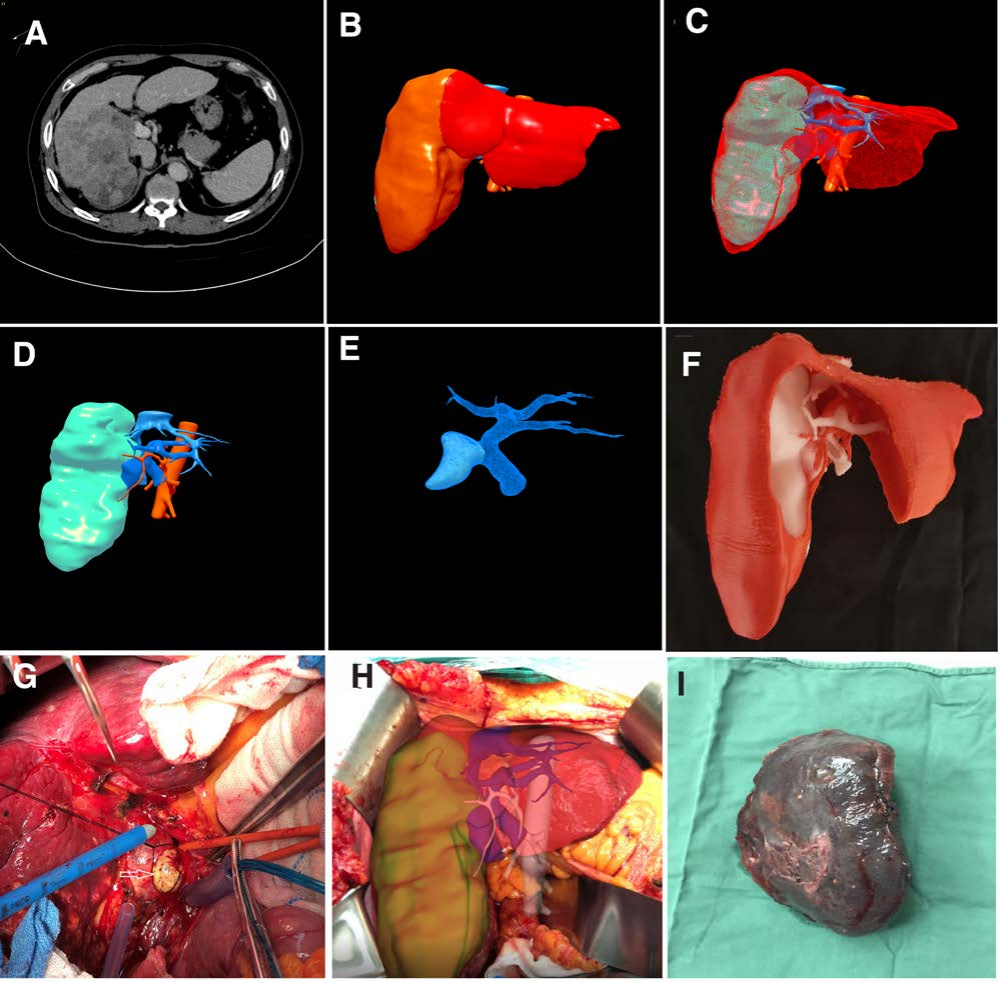
Preoperative evaluation and surgery navigation of liver cancer with portal vein tumor thrombus (PVTT). (A) The CT image showed right liver cancer with PVTT and liver fibrosis. (B) A three-dimensional (3D) reconstruction model showed the future liver remnant (FLR) was 508 mL and FLR/SLV (future liver remnant/standard liver volume) was 40.9% if right hemihepatectomy was performed. (C) Intrahepatic anatomy and tumor location Stereoscopic displayed by 3D visualization. (D) The relationship between tumor and hepatic artery, portal vein, and hepatic vein. (E) The classification of PVTT by 3D visualization (Cheng type Ⅱa or Japanese Vp classification Vp3). (F) Realistic liver anatomy with 3D printing model. The white part of the liver is composed of intrahepatic tumors, portal vein, and hepatic vein, while the red duct in the liver is the hepatic artery. (G) Portal vein tumor thrombectomy. (H) Fusion of 3D visualization image and intraoperative live scene. (I) The right liver tumor was completely removed.

We take a patient who underwent a right hemihepatectomy combined with a portal vein tumor thrombectomy as an example:

A patient whose PVTT involved the right portal vein (RPV) was accepted for surgery. 3D visualization showed the patient with Cheng type Ⅱa or Japanese Vp classification Vp3. The CT images revealed that the patient was complicated with liver fibrosis and the FLR was not enough. Preliminary assessment was inclined to non-surgical treatment. Preoperative 3D visualization showed the FLR was 508mL and FLR/SLV was 40.9% if the right hemihepatectomy was performed. Finally, the patient accepted regular right hepatectomy combined with portal vein tumor thrombectomy, and the margin was more than 1cm away from the tumor (Fig. 1).

In this study, our team performed a treatment strategy named “thrombectomy first” we proposed in 2003 (Fig. 1G), which substituted “thrombectomy first” for “liver resection first.”^[3]^ After cholecystectomy, the right hepatic artery and right hepatic duct were ligated and cut off respectively. The main portal vein (MPV), RPV, and left portal vein (LPV) were exposed by dissecting the hepatoduodenal ligament and Glisson sheath. The ultrasound further reconfirmed the patient with Cheng type Ⅱa or Japanese Vp classification Vp3. The catheter was used to suspend the MPV and LPV at the distal end of PVTT. Blocking up the MPV and LPV blood flow, followed by a cut open the anterior wall of the RPV. The PVTT was removed from the RPV incision, then opened the LPV blood flow to flush the potential residual tumor thrombus. Obstructing the LPV again, followed by opening the MPV blood flow to flush the potential residual tumor thrombus. Finally, the RPV stump was closed by 3-0 prolene continuously sutured.

The fusion of the 3D visualization model and intraoperative live scene, and the distribution of the hepatic vein and portal vein were marked on the surface of the liver to guide precise hepatectomy (Fig. 1H).

### 3.2. Results of surgical resection and perioperative complications

All operations were open surgery and performed by the same experienced physician-surgeon team. The operation plan was formulated with a 3D visualization model, which was highly consistent with the actual surgery. Among the 12 patients, 6 patients underwent right hemihepatectomy and removal of PVTT, and 6 patients underwent left hemihepatectomy and removal of PVTT. The operating time was 237.50 ± 50.23 minutes (range, 161–300 minutes), postoperative hospital stay was 15.42 ± 4.50 days (range, 8–22 days), and intraoperative blood loss was 583.33 mL (range, 100–1,700 mL). Postoperative pathological results showed that 11 cases were hepatocellular carcinoma and 1 case was intrahepatic cholangiocarcinoma. Comparison of liver function indexes on the 7th and 1st day after operation: ALT7 (71.40U/L) VS ALT1 (251.50U/L), *P <* .05; AST7 (51.79 ± 27.60 U/L) VS AST1 (400.26 ± 192.71 U/L), *P <* .05; PT7 (15.62 ± 1.52s) VS PT1 (15.56 ± 1.87s), *P* > .05; ALB7 (32.57 ± 3.70 g/L) VS ALB1 (31.18 ± 3.70 g/L), *P* > .05; TB7 (41.43 ± 26.13 μmol/L) VS TB1 (33.11 ± 18.41 μmol/L), *P* > .05.

In terms of perioperative complications: 2 patients experienced bile leakage and were cured through abdominal puncture and drainage, and 1 patient developed postoperative abdominal infection and massive ascites were treated with conservative treatment, and without procedure‑associated death.

### 3.3. Follow-up results

The follow-up data of all patients were integrity. Five patients received systemic anticancer treatment with lenvatinib or sorafenib after tumor recurrence. By the end point of follow-up, 6 patients died of tumor recurrence, 1 patient died of gastroesophageal variceal bleeding, and 1 patient died of cachexia (Table 1). The 12-month cumulative survival rate was 25.9%.

## 4. Discussion

The traditional medical imaging technology is 2D and unable to offer 3D anatomical structures of tissues or organs to surgeons, but the actual operation is accomplished in the real 3D space. Therefore, it was blindness and unreliability when the operators designed operation schemes only through 2D image data. In recent years, 3D visualization, 3D printing, and image fusion technology have been applied to liver surgery, which plays an important role in designing and guiding individualized surgery.^[4,8,10,12–15]^

### 4.1. The FLR was calculated accurately

Insufficient residual liver volume was the main cause of posthepatectomy liver failure, even death.^[16]^ Therefore, there is a need to accurately evaluate the value of FLR/SLV before operation, and it has been reported as an equally important evaluation index of liver reserve function with Child-Pugh Grade.^[17]^ Major liver resection is often required to achieve the goal of curative treatment in patients with massive liver cancer. However, the majority of patients with liver cancer are accompanied by cirrhosis caused by viral hepatitis, which is closely related to the occurrence of posthepatectomy liver failure,^[18]^ and that makes liver surgeons tend to be cautious in major liver resection. In the past, the experience of surgeons was used to estimate the FLR, which made the surgical resection margin of some patients close to the tumor and even gave up the opportunity of surgical resection. The application of 3D visualization in the measurement of residual liver volume has partly changed the present situation and made major liver resection safer.^[19–21]^ In this study, the maximum cross-sectional diameter of tumors in the selected patients was 10.15 ± 3.83 cm, and 5 patients were more than 10 cm. All patients underwent hemihepatectomy, of which 6 patients had right hemihepatectomy. The estimated value of FLR/SLV was 61.62 ± 19.38%. Compared with the first day after operation, the TB (reference value: 4–23.0 μmol/L), PT (reference value: 11–13 s) and ALB (reference value: 40–55 g/L) did not change significantly on the seventh day, while ALT (reference value: 9–50 U/L) and AST (reference value: 15–40 U/L) decreased significantly, and there was no patient suffered from postoperative liver failure. Especially for the second patient in this article, the preliminary assessment was inclined to non-surgical treatment, because the tumor diameter was 12.5 cm and complicated with cirrhosis. However, the value of FLR/SLV was 40.9 after 3D visualization and virtual right hepatectomy. Finally, the patient performed a regular right hepatectomy and recuperated successfully. These results indicate that accurate calculation of FLR/SLV was conducive to predicting the recovery of postoperative liver function and improving the safety of major liver resection. Certainly, the value of FLR/SLV could only reflect its anatomical significance. Therefore, liver cancer patients with fibrosis should also be combined with the liver reserve function (ICG: indocyanine green angiography) and the load level of hepatitis B virus DNA to estimate the risk of postoperative liver failure.^[4]^

### 4.2. Accurate classification of PVTT, and prognosis

The different types of PVTT determine the surgical plan and prognosis. The patients with Cheng type Ⅰ were suitable for en-bloc resection of the tumor with PVTT, while Cheng type Ⅳ was not recommended for surgical resection because of poor prognosis. Eastern Hepatobiliary Surgery Hospital reported 441 cases of liver cancer with PVTT the 1-year overall survival rates for Cheng types I to IV PVTT were 54.8%, 36.4%, 25.9%, and 11.1%, 3-year overall survival rates were 26.7%, 16.9%, 3.7% and 0.^[22]^ Ban et al retrospective analysis displayed 45 hepatocellular carcinoma patients with PVTT Japanese Vp classification Vp3 or Vp4(equal to Cheng type Ⅱ and Ⅲ) whose 3-year survival rates were 35.3% and 41.8%, respectively.^[23]^ Therefore, some patients could benefit from the surgery by accurate classification of PVTT and selection of appropriate cases. 3D visualization has its advantages in the classification of PVTT. Xu et al applied 3D visualization to preoperative planning of hepatocellular carcinoma with PVTT, and the results showed that 3D visualization could improve the accuracy of PVTT classification, compared with 2D CT. In addition, the prognosis of the 3D visualization group was better than the 2D CT group.^[24]^ In terms of portal vein incision and thrombectomy, our team proposed a new treatment strategy named “thrombectomy first” in 2003, which substituted “thrombectomy first” for “liver resection first.”^[3]^ The main advantages of this strategy were as follows: Crushing the tumor thrombus could be avoided during hepatectomy, which might prevent cancer cells from detaching and transferring to the remnant liver; The opening of contralateral portal venous blood flow at the early stage of surgery, which was a beneficial protective measure for the remnant liver, and helpful for recovery of liver function after operation. In our study, the accuracy of the classification of PVTT was 100% (12/12), and no patient suffered from postoperative liver failure. The patients with Cheng type Ⅱ and type Ⅲ were those who underwent hemihepatectomy combined with portal vein tumor thrombectomy, and the 12-month survival rate was 25.9%. The prognosis is similar to the literature mentioned above. In this article, there were 5 patients treated with tyrosine kinase inhibitors sorafenib or lenvatinib after tumor recurrence. We have reason to believe that the prognosis could be better if systemic anti-tumor therapy had been used early stage after operation.

### 4.3. Surgery navigation assisted by image fusion

Prejudging the distribution of intrahepatic blood vessels in the hepatectomy pathway was an important guarantee to prevent intraoperative massive hemorrhage. The disconnection of liver parenchyma no longer perplexes clinical surgeons due to the development of modern surgical technology and the emergence of new medical energy instruments, such as ultrasonic scalpels, CUSA, and so on. However, the preservation or disconnection of intrahepatic ducts has always been a difficulty during hepatectomy, especially for poorly experienced liver surgeons. There were differences in 3D spatial structures recognized by different surgeons through 2D images of the liver, which led to their inconsistent cognition of liver anatomy. Therefore, how to objectively and accurately predict the 3D spatial relationships between the distribution of intrahepatic blood vessels and tumors, which was critical for complex liver cancer in making an operative plan and guiding operation.^[8–10,25,26]^ In this study, image fusion technology was used for auxiliary hepatectomy. The fusion of the 3D visualization model and intraoperative live scene, and the distribution of the hepatic vein and portal vein were marked on the surface of the liver to guide precise hepatectomy. The operation difficulties and high-risk steps were presented in advance, which played a good “bridging” role from the virtual to the real operation.

However, the limitations of this study are: The small sample size and retrospective design, a larger prospective multicenter randomized controlled study is required to further validate the present findings. Static image fusion, the fusion image will not follow along with the liver when it is mobilized. 3D visualization and image fusion require specialists who are very familiar with the liver anatomy, and it is time-consuming, which also limits the use of other nonprofessionals.

## 5. Conclusions

3D visualization and image fusion technology could improve the accuracy of PVTT classification, formulate reasonable surgical schemes, and guide hepatectomy in real-time during operation, which is a powerful auxiliary tool for performing precise hepatectomy.

## Author contributions

**Conceptualization:** Yong Tan, Jian Yong Zhu, Cong Yun Huang.

**Data curation:** Yong Tan, Jing Li, Cong Yun Huang.

**Formal analysis:** Yong Tan, Jing Li.

**Methodology:** Yong Tan, Cong Yun Huang.

**Resources:** Yong Tan, Li Ming Wu, Zaixing Ouyang, Wen Ying Liu, Hao Song.

**Visualization:** Yong Tan, Jian Yong Zhu, Cong Yun Huang.

**Writing – original draft:** Yong Tan.

**Writing – review & editing:** Jian Yong Zhu, Cong Yun Huang.
